# Recent Advances in Engineering the Unfolded Protein Response in Recombinant Chinese Hamster Ovary Cell Lines

**DOI:** 10.3390/ijms26157189

**Published:** 2025-07-25

**Authors:** Dyllan Rives, Tara Richbourg, Sierra Gurtler, Julia Martone, Mark A. Blenner

**Affiliations:** 1 Department of Chemical & Biomolecular Engineering, Clemson University, 206 S. Palmetto Blvd., Clemson, SC 29634, USA; dyllanmrives@gmail.com (D.R.); sgurtler@email.sc.edu (S.G.);; 2 Department of Chemical & Biomolecular Engineering, University of Delaware, 590 Avenue 1743, Newark, DE 19713, USA

**Keywords:** Chinese hamster ovary (CHO) cells, ER stress, unfolded protein response (UPR), therapeutic proteins, biopharmaceuticals, synthetic biology

## Abstract

Chinese hamster ovary (CHO) cells are the most common protein production platform for glycosylated biopharmaceuticals due to their relatively efficient secretion systems, post-translational modification (PTM) machinery, and quality control mechanisms. However, high productivity and titer demands can overburden these processes. In particular, the endoplasmic reticulum (ER) can become overwhelmed with misfolded proteins, triggering the unfolded protein response (UPR) as evidence of ER stress. The UPR increases the expression of multiple genes/proteins, which are beneficial to protein folding and secretion. However, if the stressed ER cannot return to a state of homeostasis, a prolonged UPR results in apoptosis. Because ER stress poses a substantial bottleneck for secreting protein therapeutics, CHO cells are both selected for and engineered to improve high-quality protein production through optimized UPR and ER stress management. This is vital for optimizing industrial CHO cell fermentation. This review begins with an overview of common ER-stress related markers. Next, the optimal UPR profile of high-producing CHO cells is discussed followed by the context-dependency of a UPR profile for any given recombinant CHO cell line. Recent efforts to control and engineer ER stress-related responses in CHO cell lines through the use of various bioprocess operations and activation/inhibition strategies are elucidated. Finally, this review concludes with a discussion on future directions for engineering the CHO cell UPR.

## 1. Introduction

Biopharmaceuticals continue to be one of the fastest growing segments of the pharmaceutical industry. As such, the global market for therapeutic proteins, such as monoclonal and polyclonal antibodies (mAbs and pAbs, respectively), is expected to grow to USD 679 billion by 2033 [1]. Chinese hamster ovary (CHO) cells are the most common host cell used for biopharmaceutical and therapeutic protein production [2,3]. These host cells provide benefits in the industry such as human-like post-translational modifications (PTMs), efficient secretion systems, a well-developed safety profile, and suspension adaptability [3,4]. In fact, at least 76 CHO-derived therapeutics have been approved by the Food and Drug Administration between 2020 and 2024 [5]. While CHO cells have the potential to meet all production needs, research efforts continue to work towards cellular process optimization to achieve higher titer, higher productivity, and consistent product quality attributes. These outcomes are strongly influenced by protein processing within the endoplasmic reticulum (ER) and the unfolded protein response (UPR).

Preferred analytical methods for studying ER stress have been elucidated elsewhere [6,7,8]. Other reviews have highlighted various strategies for improving recombinant protein production in CHO cells, and some, if not all, of these strategies have associations with ER stress [9,10,11,12,13,14,15,16,17]. This review comprehensively outlines various UPR-focused engineering strategies and the corresponding effects on CHO cell bio-production as described in the recent literature. For context, this review briefly summarizes how the UPR and other downstream pathways are activated and utilized to overcome ER stress. Common UPR-related markers are identified, and recent findings characterizing the UPR in recombinant CHO cells are elucidated. Next, strategies for manipulating the UPR are separated into bioprocess choice, culture conditions, and cell line development. Finally, this review concludes with our thoughts on the future directions for engineering the CHO cell UPR. Studies focused on other cell signaling pathways are considered outside the scope of this review. Research using only non-producing CHO cell lines is also considered outside the scope of this review.

## 2. An Overview of the UPR

**Figure 1:**
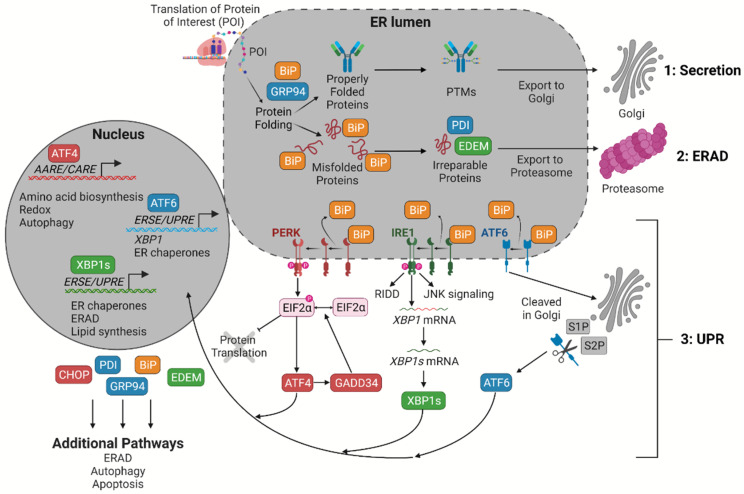
Accumulation of unfolded proteins results in the UPR. After translation, proteins that are properly folded are secreted (route 1: Secretion), while misfolded proteins are broken down in order to recycle important amino acids for continued production of other proteins (route 2: ERAD). Stress in the ER occurs when misfolded proteins accumulate. When the chaperone BIP binds misfolded proteins, a downstream transcription cascade (route 3: UPR) is initiated to either relieve burdens on the ER or activate apoptotic pathways if the former cannot be achieved. Italics within the nucleus represent the promoter elements bound by each transcription factor. Previously undefined abbreviations: IRE1-dependent decay (RIDD); site-1 and site-2 proteases (S1P and S2P, respectively). Figure created with BioRender.com.

Industrial recombinant CHO cell lines can suffer from a high burden on the ER due to secretion and titer requirements coinciding with increased productivity and product quality demands [14,18,19,20]. The ER is the organelle primarily responsible for secreted protein synthesis owing to its anchored ribosomes carrying out protein translation and a distinct set of resident proteins, so-called ER resident proteins, continuously facilitating proper protein structure and folding. There are three main outcomes for proteins produced within the ER (Figure 1) [21,22]. In the first route, properly folded proteins receive PTMs prior to exiting to the Golgi. In the second route, referred to as ER-associated degradation (ERAD), misfolded proteins are marked as irreparable by ubiquitination and are digested by the proteasome. In the proteasome, the amino acids are recycled to make new proteins [23]. In the third route, accumulation of misfolded proteins within the lumen of the ER, referred to as ER stress, results in multiple signaling responses collectively called the unfolded protein response (UPR).

In CHO cells, the UPR has three main pathways delineated by an initiator protein, either cyclic adenosine monophosphate (cAMP)-dependent transcription factor 6 (ATF6), inositol-requiring endoribonuclease 1 (IRE1), or protein kinase R (PKR)-like endoplasmic reticulum kinase (PERK) [24,25,26]. During homeostasis, each of the initiator proteins is bound by the chaperone glucose-regulated protein (GRP) 78, commonly referred to as binding immunoglobulin protein (BIP). The chaperone BIP resides in the ER lumen and participates in protein folding, binding, and transport across the ER membrane. Each of the UPRs is initiated after the BIP’s dissociation from the initiator protein and preferential binding to luminal unfolded proteins [24,25,26,27]. The signaling cascades resulting from UPR activation serve as major quality control mechanisms within mammalian cells. The primary outcome of each UPR pathway is activation of one of three transcription factors that orchestrates a coordinated multifaceted response (ATF6α, spliced X box-binding protein 1 (XBP1s) and cAMP-dependent transcription factor 4 (ATF4)). The corresponding increases in pathway-specific gene expression (e.g., amino acid biosynthesis, lipid synthesis, ER expansion, ERAD, and protein processing) are aimed at ameliorating stress on the ER, increasing protein secretion, and preventing chronic stress and apoptosis. There is significant crosstalk between UPR pathways since many UPR target genes contain one or more of the same promoter elements (e.g., ERSE I, ERSE II, and C/EBF-ATF, etc.) needed for transcription factor binding. The UPR pathways and the crosstalk between them is important for improving secretion and productivity of recombinant CHO cell lines. The extent of ER stress is typically assessed by comprehensive measurement of multiple genes and proteins that may be directly or indirectly related to the UPR. Specific details for UPR pathways and crosstalk have been well documented, and common markers used for the remainder of this review are summarized in Table 1 [6,7,8,14,24,26,28,29,30,31,32,33,34,35,36,37,38,39,40,41,42,43,44,45,46,47,48].

## 3. An Optimized UPR Is Necessary for High-Producing CHO Cell Lines

Contrary to the notion that ER stress opposes protein production, many outcomes of the UPR are beneficial for protein production such as increased expression of chaperones, foldases, and trafficking proteins. Indeed, researchers report that high producers have an enhanced UPR profile in comparison to their lower-producing counterparts [18,25,49]. For two different products (2F5- and 3D6-scFv-Fc), one group tested three different transgene delivery methods and found that cell lines producing 3D6-scFv-Fc consistently exhibited higher fold differences in specific productivity. Proteins involved in protein folding such as PDIA3, CRT, PDIA4, and GRP94 were also found to be enriched in these producers [18]. A direct comparison of high and low IgG producers during batch culture resulted in increased expressions of *BIP*, *GRP94*, *CNX*, *CRT*, *ERDJ4*, *ATF4*, *CHOP*, *GADD34*, *NRF2*, and *XBP1s* in the high producer [25]. Transfected subclones of two different host lines also upregulated *BIP*, *GRP94*, *PDIA3*, *CHOP*, *ATF4*, *HERPUD1*, and other genes involved in ERAD, indicating that these markers are expressed with increasing productivity of IgG [49].

**Figure 2:**
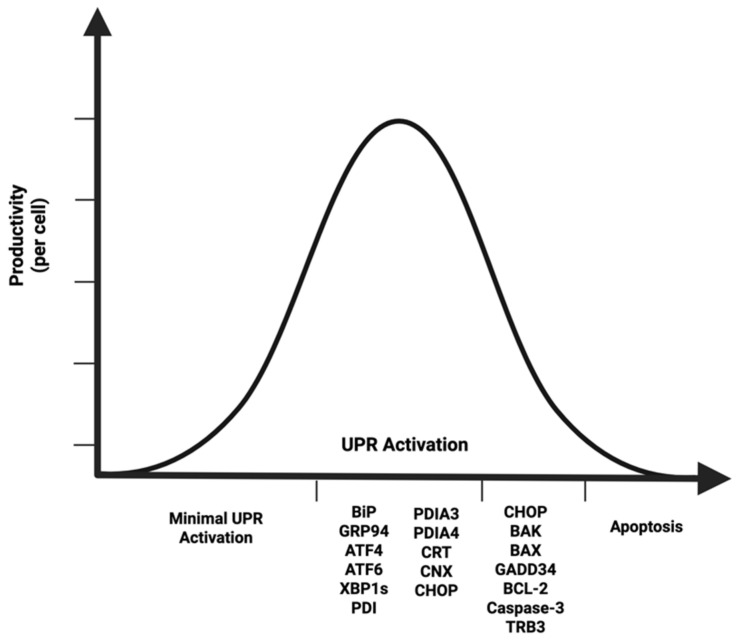
UPR activation is an optimization problem in CHO cell line development. A moderate amount of ER stress is advantageous for high productivity. If the UPR is only minimally activated, the cell line will exhibit low productivity. Likewise, if the cell line has an overactive UPR, it might exhibit low productivity due to apoptosis. Figure created with BioRender.com.

There are also a plethora of studies reporting positive results from multiple UPR-related engineering strategies (see Section 6.2), but these strategies do not always yield positive results. On the other hand, there are also studies that report a minimally activated UPR [50,51]. Collectively, this supports the need for an optimized UPR profile in recombinant CHO cells. Thus, because a high workload is created by recombinant protein production, increased expression of UPR biomarkers is expected in high-producing (HP) CHO cells, but both a minimally activated UPR and an overly active UPR can lead to negative outcomes such as low productivity (LP) and apoptosis, conceptually shown in Figure 2.

## 4. The Context Dependency of UPR Engineering

Given the role the UPR plays in protein folding and secretion, the optimal UPR profile for any given CHO cell line is likewise impacted by the same variables affecting recombinant protein production. Recombinant proteins are designated easy-to-express (ETE) or difficult-to-express (DTE). Compare, for example, the structural differences between IgG_1_-type mAb products, bi-specific antibodies (antibodies that bind two antigens, BsAbs), and multi-specific antibodies (antibodies that bind multiple antigens, msAbs) where the structure requirements are highly dependent on the correct pairing of multiple subunits [52]. Specific components of a protein’s structure (e.g., disulfide bonds, post-translational glycans, etc.) are also variables since protein production and folding rely on sufficient nutrients (e.g., amino acids), calcium-dependent chaperones, and redox power for disulfide formation [43]. Table 2 reports the observed UPR profile of recombinant CHO cell lines on a product-specific basis.

With respect to the secretory capacity of cellular machinery, a cell line’s expression level of the product (recombinant protein load) is another factor in UPR activation [77]. Host cell line specifics, selection methods applied for a recombinant cell line, and bioprocess parameters are additional variables with impacts on product yields, quality, and UPR activation. The next sections of this review discuss optimizing the UPR through bioprocess choice, culture conditions, and cell line development.

## 5. Bioreactor Operations Elicit Different ER Stress Responses

### 5.1. Batch Processes

Batch processes are a suitable standard for comparing multiple engineering strategies, while increased volumetric productivities and product yields generally require fed-batch or perfusion processes. Batch processes provide ease of setup; however, many stressors are also introduced, such as nutrient depletion, osmotic/oxidative stress, lactate/ammonia buildup, pH increases, etc. [72,78]. Productivity can remain high during the exponential phase of cultures, but the death or decline phase of these cultures shifts the UPR dynamic to pro-apoptotic marker expression [72]. Chaperones *BIP*, *GRP94*, *PDI*, and the transcription factor *ATF4* were upregulated in EPO-producing cells when unstressed, but, during the death phase, other markers were also expressed including *CHOP*, *Trb3*, *Odz4*, *Sqstm1*, *Sels*, and *HERPUD1*. Despite tunicamycin-induced adaptability to ER stress, these results are somewhat mirrored in a batch culture of anti-rhesus D IgG-producing CHO cells, which exhibited increased expression of *XBP1s*, *BIP*, *CRT*, and *CHOP* [78]. As observed from both studies, late batch culture induces the PERK pathway. While the PERK pathway is known for increasing amino acid biosynthesis, pro-apoptotic markers such as CHOP and Trb3 are typically activated as end results of a prolonged UPR.

### 5.2. Fed-Batch and Perfusion Processes

Fed-batch and perfusion processes are better for improving titers and productivities by circumventing the pro-apoptotic impacts of the PERK pathway. Fed-batch conditions are less nutrient-limited than batch conditions and can contribute to higher productivities as observed when there was an increase of 50 pcd for ER stress-adapted cells compared to 25 pcd for control, non-adapted cells [78]. Media recycling in perfusion processes extends nutrient availability even further. Two different research groups compared fed-batch and perfusion processes for culturing bsAbs-producing CHO cell lines [75,76]. In the first study reduced product aggregates were observed after using a perfusion process. Expressions of BIP, CHOP, and ATF6 as well as specific productivity were increased in the fed-batch process but decreased in the perfusion process. This group also observed no differences in PDI expression between fed-batch and perfusion processes [75]. In contrast, the second group reported increased product aggregates in the perfusion process despite similar BIP and ATF6 expression results (i.e., BIP and ATF6 expressions were increased in the fed-batch processes). In the latter study, an ER pH sensor was developed based on *CRT* sequences, and pH measurement was concluded to be a better indicator for aggregate formation possibly because pH can impact the protein folding environment of the ER [76]. Both studies report increased expressions of BIP and ATF6 coinciding with bsAb production. These results suggest that perfusion culturing of a bsAb producer with an enhanced UPR profile may have positive effects on product aggregation and productivity.

### 5.3. Feeds

The need for increasing titers of recombinant therapeutic proteins requires maintaining healthy productive cultures, a nutrient-demanding endeavor. Biomarkers of the UPR are useful indicators for culture health, longevity, and productivity. Recent studies have investigated changes in UPR activation after altered levels of key nutrients with both saturation and depletion causing negative effects. Hyperosmolality feeding conditions induced expression of multiple UPR markers, primarily heat shock proteins and chaperones [79]. Saturated glucose increased specific productivity at the expense of decreased IVCD and increased cell death [80]. This condition increased expressions of *NCK1*, *HtrA2*, and calpains while downregulating *PRKRA*. Cysteine is another important nutrient because of its role in disulfide bond formation [81,82]. Excessive cysteine results in increased expressions of *IRE1α/β*, *ATF6α/β*, *ATF4*, *CHOP*, *ATF3*, *HSP70*, *HSP40*, *UBXN4*, *GADD34*, and *ERDJ4* [83], while low cysteine feed conditions induce expressions of *BIP*, *CHOP*, *BCL2L11*, *IRE1*, ERO1α, GRP94, *GADD34*, *BECN1*, and *ATF3* [81,82]. Based on these studies, saturation and depletion of cysteine overwhelm cellular capacity, resulting in the activation of all three UPR arms. Changes in media, feeds, and feed timing resulted in the increased expression of chaperones BIP and PDI, with the latter positively correlating with productivity increases in a mAb [84]. This study illustrates the importance of nutrient maintenance for optimum UPR activation and high productivity.

### 5.4. Temperature Downshift

**Figure 3:**
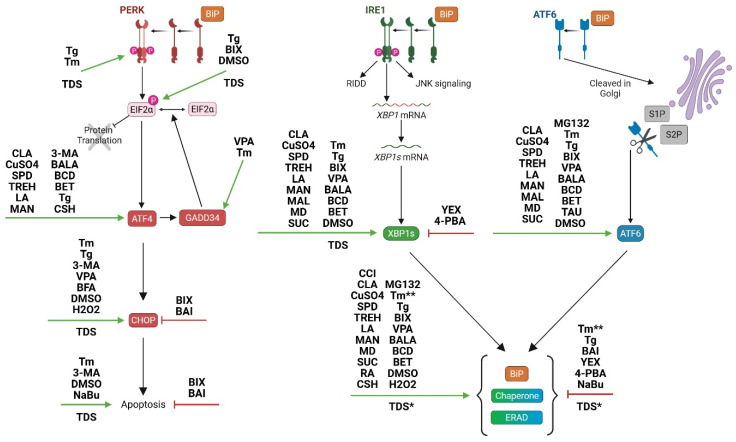
Effects of reduced temperature (Section 5.4) and chemical treatments (Section 6.1) on UPR activation. The UPR pathways are the same as shown in Figure 1. The PERK pathway is indicated in red, the IRE1 pathway is indicated in green, and the ATF6 pathway is indicated in blue. Activation of a UPR biomarker is indicated by a green arrow. Inhibition of a UPR biomarker is indicated by a blocked red line. The effects of temperature downshift are indicated as TDS. The effects of chemical treatments are shown using their respective abbreviations, which are as follows: 3-methyladenine (3-MA); baicalein (BAI); beta alanine (BALA); beta cyclodextrin (BCD); betaine (BET); BIP inducer X (BIX); thapsigargin (Tg); tunicamycin (Tm); valproic acid (VPA); yeast extract (YEX); copper sulfate (CuSO4); spermidine (SPD); trehalose (TREH); linoleic acid (LA); conjugated linoleic acid (CLA); mannose (MAN); cottonseed hydrolysate (CSH); maltose (MAL); maltodextrin (MD), sucrose (SUC); proteasome inhibitor MG132 (MG132); taurine (TAU), dimethyl sulfoxide (DMSO); hydrogen peroxide (H2O2); sodium butyrate (NaBu); cell cycle inhibitor (CCI); and rosmarinic acid (RA). * The use of TDS causes increases in *HERPUD1* for rh-tPA [90] and decreases in *HERPUD1* for EPO-Fc [92]. ** Sulaj et al. report downregulation of BIP and PDIA4 in response to Tm [56]. Figure created with BioRender.com.

Reducing CHO cell culture temperature enables better protein folding, and many processes utilize temperature downshift (TDS) [19,85,86,87,88,89] to improve recombinant protein yields [90]. Implementing TDS has been shown to increase *MYC* expression, a transcription factor involved in growth and the cell cycle [90,91]. Under mild hypothermia, the increase in *MYC* expression also coincides with increased *XBP1s* expression [91,92]. Chaperones activated downstream of XBP1s have also been reported as increased during TDS including *PDI*, *PDIA3*, *BIP*, *CRT*, *CNX*, and *GRP94* [90,92,93]. Additionally, multiple markers of the PERK and ERAD pathways have been reported as upregulated during TDS including *PERK*, *ATF4*, unphosphorylated and phosphorylated eif2a, *CHOP*, *Trb3*, *HERPUD1*, *UGGT2*, *ERLEC1*, and *Sec31b* [90,92,94,95], although decreased expression of *EDEM3*, *SELS*, *HERPUD1*, and *SYVN1* has also been reported after TDS [92]. The PERK and ERAD pathways have roles in amino acid synthesis and ER quality control [23]. Dynamic expression changes in chaperones and the PERK/ERAD pathways are to be expected since TDS has an effect on protein folding kinetics [94]. The reported effects of TDS on UPR activation are illustrated in Figure 3.

## 6. Controlling the UPR Using Chemical Additives and Cell Line Development

### 6.1. Chemical Additives

Well known chemicals such as tunicamycin (Tm), thapsigargin (Tg), dithiothreitol (DTT), or brefeldin A (BFA) have specifically defined modes of action for inducing ER stress by interfering with N-glycosylation, calcium influx into the ER, disulfide-bond formation, and protein transport to the Golgi, respectively [6]. There are many other chemical additives with multiple applications for recombinant CHO cell lines including use as positive ER stress controls, tools for the identification of engineering targets, tools for the selection of high-productivity clones, and chemical chaperones during bio-production. For example, one study found the upregulation of *XBP1s* and multiple genes in the Hexosamine Biosynthetic pathway (HBP) pathway in response to Tm-induced ER stress adaptability (i.e., impaired glycosylation) [78]. The impacts of these chemical additives as reported in the recent literature are illustrated in Figure 3 [56,67,72,78,89,96,97,98,99,100,101,102,103,104,105,106,107,108,109].

### 6.2. Cell Line Development

**Figure 4:**
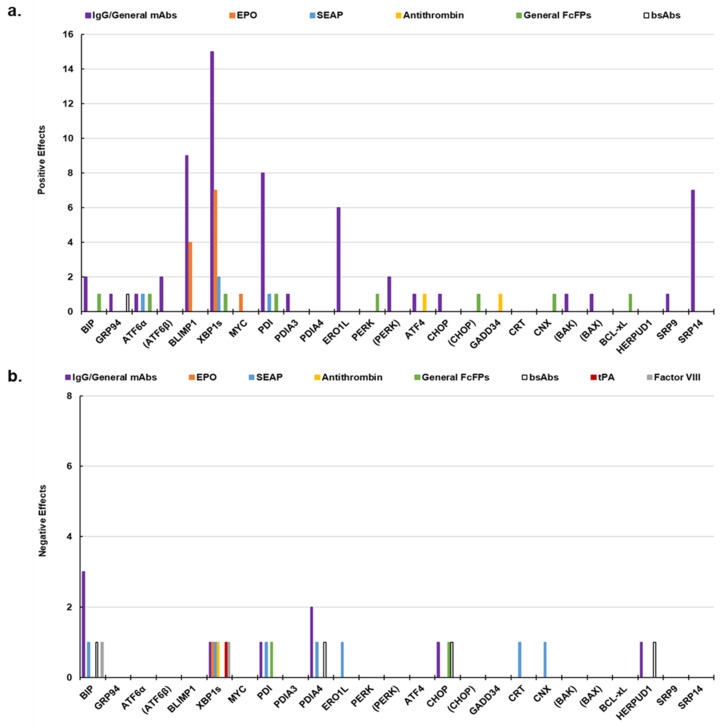
Positive and negative effects of CHO cell UPR engineering for different products. Based on Table 3. (**a**) Positive effects include increased titer, yield, and q_p_, etc. (**b**) Negative effects include decreased titer, yield, q_p_, etc. UPR targets shown in parentheses are downregulated or knocked out. The total number of positive/negative effects shown on the y-axis for each UPR target includes co-expression studies. The General mAbs category includes ETE Trastuzumab (Tras), DTE Infliximab (Infli), humAb 2F5 IgG, anti-IL-8 IgG, TfR-Ab, DTE Doppelmab, Adalimumab, ETE rituximab, and hTRA-8. The EPO category includes EPO-Fc. The General FcFPs category includes TNFR-Fc and DTE Sp35Fc.

It is important to note the lack of a universal engineering strategy for improving titers or productivity. As discussed in Section 4, the success, or lack thereof, of any given UPR engineering strategy is dependent on many factors. For example, increasing expression of *XBP1s* typically increases production of mAbs [88,110,111], but the result is not repeated with other protein products such as Antithrombin III (AT-III) [112], Human Factor VIII [113], or tissue plasminogen activator (t-PA) [114,115] (see Table 3 and Figure 4). There has been recent success with overexpressing *BLIMP1* and/or *XBP1s*, which are both observed to play key roles in antibody production in plasma cells, professional antibody-secreting cells [85,116,117,118,119,120]. As another example, downregulating *PERK* increased titer and productivity for two different mAb producers, but upregulating *PERK* decreased product aggregates in a TNFR-Fc producer [94,121]. These results suggest the UPR engineering strategy utilized may be dependent on the cell line and product. The expression level of the recombinant protein and whether it saturates the secretory capacity of the cell is another key factor [77,122]. The expression level of one or more UPR biomarkers must also be considered, and many researchers have studied the impacts of co-expression of multiple UPR biomarkers on bio-production [20,77,86,87,110,111,118,122,123,124,125,126,127,128,129,130]. While multiple researchers report unaffected or improved product quality following manipulation of the UPR, many simply do not report the effects on product quality. The impacts on product quality as a result of manipulating expression of UPR biomarkers should not be taken lightly. The effects of engineering expression of UPR-related biomarkers in recombinant CHO cell lines are summarized in Table 3. Figure 4 presents general findings of Table 3 on a product-specific basis.

Some researchers have also applied the use of long non-coding RNAs (lncRNAs) or microRNAs (miRNAs) for controlling the expression of multiple UPR markers [28,49,146,151,152,153]. Other noteworthy research focuses on utilizing UPR biomarkers as reporters or sensors for isolating high-productivity cell lines and the monitoring of culture production [76,154,155,156,157,158,159]. The sequence and promoter elements of UPR biomarkers have been applied in novel approaches such as the pH sensor developed using *CRT* sequences and the *BIP* promoter element used to increase production of IgG_1_, IgG_2_, and IgG_4_Pro [76,155]. Another study integrated *GFP* into the *BIP* promoter and observed increased fluorescence, titer, and productivity in mAb-producing cells [156]. Two similar studies developed UPR-induced reporters based on *GFP* expression utilizing the ER/UPR promoter elements ERSE and UPRE, although the first study also used the amino acid response element AARE; the *BIP*, *CRT*, and *GRP94* promoters; and the *XBP1* intron sequence [154,159]. The first study found the *BIP* promoter construct to be the best indicator of IgG-producing CHO cells [154]. The second study focused on ATF6α and XBP1s activation using both ERSE (preferential binding by either ATF6α or XBP1s) and UPRE as well as the ACGT core element (preferential binding by XBP1s) to monitor GFP expression/UPR induction during production. Cultures with the highest induction of UPRs showed improved production performance [159]. A dual fluorescent reporter system was developed through expression of *Red fluorescent protein (RFP)-XBP1-GFP* fusion [157]. In the absence of XBP1s activation, only *RFP* was expressed; conversely, when *XBP1* is spliced, *GFP* was placed in the same reading frame as RFP, resulting in RFP and GFP-positive cells. An ER stress index (ERSI) was created to quantify ER stress using the ratio between cells expressing both GFP and RFP and cells only expressing RFP. Three different IgG-producing cell lines with different productivities were tested, and the cell line achieving the highest titer of >5 g/L exhibited the highest ERSI at a ratio of 1.0 by late fed-batch. The reporter was also tested during cell line development where 42% of clones with titers > 1 g/L exhibited a high ERSI > 0.2. Given the importance of BIP and XBP1s, these types of reporters will be very useful in the selection of high-producing cell lines, particularly those producing DTE products. As a final note, multiple research groups have succeeded in improving titers and productivity by applying combinations of strategies that include changing the bioprocess type, applying a TDS, adding chemical modulators, and manipulating expression level ratios of recombinant protein and UPR biomarkers [19,85,86,87,88,89].

## 7. Future Directions

The combined use of molecular biophysical models and ER stress sensors holds promise for determining the critical structural features of proteins that activate specific pathways of the UPR. Formalizing the meaningful parameters of protein-specific ER stress may be more practical than trying to find the structural features that lead to specific ER stress responses. For example, the measurement of the ER’s capacity to express an arbitrary protein relative to the onset of UPR induction may provide more actionable insights. A broad meta-analysis of the existing CHO transcriptomic and proteomic data sets may yield insights into complex non-linear relationships between the ER state and the ER stress response. Likewise, expression ratios of UPR transcription factors and product mRNAs will need to be studied in order to optimize the UPR profile for any given cell line.

There are many recent reports of success in improving titers and productivity of mAbs using the overexpression of *BLIMP1*, a transcription factor found to induce *XBP1s* expression in plasma cells, which are professional antibody-secreting cells. Similarly, for the production of other recombinant proteins (EPO, t-PA, IFN, etc.), transcription factors and other UPR markers specifically induced in the native environment should be explored. Using ER stress-inducing chemicals for adaptation during cell line development is a promising strategy. Depending on the specific requirements of any given recombinant product, different chemicals or stress conditions should be studied in adaptation strategies (e.g., DTT, Tg, BFA, reduced glucose, etc.).

Perfusion processes circumvent the proapoptotic impacts of the UPR, and we think that combining perfusion culture with CHO cell lines with stress adaptability and/or UPR strategies, as reported in Table 3, might increase productivities and yields even further. Inducing the downregulation of cell cycle genes in order to cause G0/G1 arrest at maximum VCD, combined with inducing the upregulation of UPR transcription factors, may be applied in order to shift resources from growth to protein production. This endeavor may be aided by using promoters sensitive to environmental factors (e.g., light, temperature, or pH) rather than chemically induced promoters. Another future direction is the further exploration of product quality attributes in response to ER stress induced by high specific productivity. Research would benefit the biopharmaceutical industry and the field by ensuring that product quality is unaffected or improved by any given UPR engineering strategy. Finally, we expect to see the increased use of single and multiple ER stress modulating genetic targets incorporated into cell lines prior to cell line development.

## Figures and Tables

**Table 1:** Common ER stress markers.

Marker *	Role
**HSPA5**/**GRP78**/**BIP**	UPR initiator; chaperone
**HSP90B1**/**GRP94**	Chaperone
ATF6c/ATF6α	UPR initiator; transcription factor
ERN1/IRE1	UPR initiator; endoribonuclease
*XBP1s*	Transcription factor
**P4HB**/**ERP59**/**PDIA1**/**PDI**	Isomerase; chaperone
**ERP57**/**PDIA3**	Isomerase; chaperone
**ERP72**/**PDIA4**	Isomerase; chaperone
**ERO1L**	ER oxidoreductase
JNK	Kinase
PERK	UPR initiator; kinase
EIF2α	Translation
**ATF4**	Transcription factor
**GADD153**/**DDIT3**/**CHOP**	Transcription factor
**PPP1R15A**/**GADD34**	Translation initiation; apoptosis
*EDEM1*, *EDEM2*, *EDEM3*	ERAD; mannosidases
*DERL2*, *DERL3*	ERAD
*HSPA8*	Heat shock protein; chaperone; ERAD
*HSP70*	Heat shock protein; chaperone
*CALR*/*CRT*	Calcium-dependent chaperone
**CANX**/**CNX**	Calcium-dependent chaperone
*BAK*	Apoptosis
*BAX*	Apoptosis
*BCL2*	Apoptosis
Caspase-3	Apoptosis
*TRB3*	Apoptosis
*HERPUD1*	ERAD
*HYOU1*	Hypoxia

* Underlined names are used for reference throughout this review for markers with multiple indications. Measurement of a UPR marker is dependent on whether the marker is activated transcriptionally or post-translationally. Markers in italics are typically measured as mRNA. Markers in bold can be measured as mRNA or protein. Otherwise, markers are measured as protein.

**Table 2:** UPR profile exhibited by various recombinant CHO cell lines.

Product	Markers Identified by Omics/Profiling *	Reference
IgG_1_ **	CHOP, ATF4, BIP, GRP94, HERPUD1, PDIA3, BCL-XL, PRDX1, USP14, SOD1, SOD2, BCL2L11, PDIA4, PDI, PDIA6, RAGC, RPN1, CRT, CNX, ERDJ4, ERO1α, XBP1s, UGGT1-V1, UGGT2, GADD34, NRF_2_, HYOU1, SIL1, DNAJC1, DNAJC3, DNAJC10, DNJC11, FKBP9, HSPE1, PRDX1, (CREDL1), (SELENBP1)	[25,49,50,53,54,55,56,57,58,59]
IgG2	ATF4, BIP, RAGC, RPN1, CHAC1, DERL3, HSP70 CRT, HERPUD1, HSPA9, RAGC, RPN1	[53]
IgG4	UGGT1, HSP90AB1, WFS1, GRP94, BIP, HYOU1, PDIA5, PDIA4, ERP29	[60]
IgM	-	[51]
General mAbs ***	(PDIA3) FK506-binding proteins 7 and 14, calumenin, NCK1, PRKRA, BIP, PERK, CHOP, ATF6, XBP1s, PDI, GRP94, PDIA4, CNX, SEC61, HSP90, DNAJB9, DNAJB11, PDIA2, PDIA3, EDEM1, EDEM3, UGGT1, KDELR1, (CLCC1), (DNAJC3), (EMC7), (OS9), (MINPP1), (TMED4), (UFC1), (PRKCD), (PITPNM1), (SURF4)	[61,62,63,64,65,66]
tPA	HSPA8	[67]
Factor VIII	BIP, XBP1s, CRT, CNX, PDIA3, PDIA4, PDIA6, EDEM1, EDEM2, DERL2, HERPUD1, PRDX1	[68,69]
Antithrombin (AT(C95R))	BIP, GRP97, PDI	[70,71]
EPO	CHKB, CHKA, CEPT, HERPUD1, SYVN1, SELS, EDEM3, SQSTM1, XBP1, PDI, GRP94, BIP, BIRC5, ODZ4, ERO1L, TRB3, CHOP, ATF5, ATF4	[72,73]
General FcFPs ****	Cathepsin B, PDIA3, CRT, PDIA4, DNAJC7, PDI, PDIA6, GRP94, GRPLE1, p-EIF2α, EI5FA, EIF4A1, XBP1s, BIP, PRDX1, CAT, HSP90AB1	[18,74]
bsAbs	BIP, ATF6, PDI, PERK, CHOP	[66,75,76]
tsAbs	(PDI), (DNAJA3), (DNAJC1), (XBP1s), (ATF4), (ATF6), (CEBPA), (CEBPB), (CEBPD), (CEBPG), (IRE1), (INSIG1), (MAP2K7), (MAPK8), (NRF2), (PDI), (ATF5), (RPL28), (SCAP), (SREBF1), (NUPR1), (UBXN4) CEBPZ, DNAJC7, DNAJC21, HSPA9	[52]

* Downregulated/knockdown are indicated in parentheses. ** Papers with IgG-producing lines are assumed as IgG_1_ if not otherwise stated. *** This category includes anti-CD20, anti-CEA, Trastuzumab (Tras), Infliximab (Infli), anti-TNF. **** This category includes Sp35Fc, 3D6-scFv-Fc, 2F5-scFv-Fc, hCD200Fc.

**Table 3:** UPR biomarker expression studies and effects on recombinant production.

Target *	Cell Line **	Recombinant Product	Effects ***	Impact on Quality (Y/N/U) ****	Reference
XBP1s	DG44	IgG	Increased yield, q_p_	N	[110]
XBP1s/XIAP	DG44	IgG	Increased yield, q_p_	N	[110,123]
ERP27	CHO-K1d	ETE Trastuzumab (Tras)	Increased titer	U	[124]
ERP27/PDIA3		DTE Infliximab (Infli)	Increased titer, VCD, viability	U	
ERP27/PDIA3		DTE Etanercept	Increased titer, VCD, viability	U	
(PERK)	CHO-K1	mAb2	Increased titer, q_p_, decreased viability	N	[121]
(PERK/Bax/Bak)	CHO-K1	mAb3	Increased titer, q_p_, IVCC, viability	N	
(ATF6β)	DG44	IgG	Increased titer, VCD	N	[28]
(ATF6β)	CHO-K1d	IgG_1_	Decreased VCD, no change in titer, increased q_p_	U	[131]
(WFS1)			Decreased titer, no change in growth	U	
BIP	CHO DHFR-	humAb 2F5 IgG	Decreased production rate	U	[125]
PDI			Increased production rate	U	
BIP/PDI			Decreased production rate	U	
XBP1s	CHO-S	Multiple mAbs	Increased mAb expression levels	U	[111]
ERO1a	CHO-K1	Multiple mAbs	Increased mAb expression levels	U	
XBP1s/ERO1a	CHO-S	Multiple mAbs	Increased mAb titers	N	
XBP1s	CHO-K1	Human Factor VIII	No improvement in production	U	[113]
XBP1s	CHO-K1	Tissue Plasminogen Activator (t-Pa)	No improvement in titer	U	[114,115]
PDI	CHO-DUKX B-11	TNFR:Fc	Decreased secretion	U	[132]
PDI		IL-15	None	U	
BIP	CHO-DUKX B-11	von Willebrand Factor	Decreased secretion	U	[27]
BIP		Mutant Factor VIII	Decreased secretion	U	
BIP		M-CSF	None	U	
eIF3c	CHO-K1	cap- and IRES-Dependent Recombinant Protein	Improved recombinant protein synthesis, cell count	U	[133]
XBP1s	CHO-K1	IgG	Increased q_p_, ER size	N	[88]
ATF4	CHO-DP12 SF	anti-IL-8 IgG	Increased q_p_	U	[134]
BIP	-	TfR-Ab	Increased titer, viability	N	[135]
(PDIA4)	CHO-HcD6 (CHO-K1d)	ETE Trastuzumab (Tras)	Decrease in secreted antibody	U	[136]
PDIA4			None	U	
XBP1s	CHO DG44	mAb	No improvement in titer	U	[77]
	CHO DHFR-	Interferon γ (IFNγ)	No improvement in titer	U	
	CHO-K1	EPO	No improvement in titer	U	
XBP1s	CHO-K1	EPO	Increase in titer is dependent on product/XBP1s dosage levels	U	[77,122]
(XBP1s)			Decreased product titer	U	
MYC	CHO-K1d	EPO	Increased IVCC	U	[87]
XBP1S			Increased titer, q_p_	U	
MYC/XBP1s			Increased IVCC, specific growth rate, titer, q_p_	U	
PDI	-	Thrombopoietin (TPO)	No increase in q_p_	U	[126]
	CHO DG44	mAb	Slight increased q_p_	U	
BLIMP1	DG44	mAb	Increased titer, q_p_	U	[116]
DNAJC3				U	
SYVN1				U	
SELENOF				U	
HSPA8				U	
BLIMP1	CHO-K1	IgG and DTE Doppelmab	Increased titer	U	
SYVN1				U	
DNAJC3				U	
ATF4	CHO DXB11	Antithrombin III (AT-III)	Increased q_p_	U	[112]
XBP1s			No improvement in q_p_	U	
GADD34	CHO DXB11	Antithrombin III (AT-III)	Decreased VCD, Increased q_p_	N	[137]
BCL-xL	CHO DG44	Fusion Protein (FP)	Increased q_p_	N	[138]
NFKBIZ	CHO-HcD6	IgG_1_	Increased q_p_	N	[139]
PDI/XBP1s	CHO-S	Adalimumab	Increased titer, q_p_	U	[86]
		SEAP	Increased product expression	U	
KDEL receptor 1	CHO-K1	IgG	Increased q_p_	N	[140]
BLIMP1	CHO-K1	IgG_1_	Decreased VCDs, prolonged viability, Increased titers, q_p_	U	[85,117,118]
		EPO-Fc		U	
	CHO-S	IgG_1_		U	
BLIMP1	CHO-K1	EPO-Fc	Decreased VCD, increased titer, q_p_	U	
	CHO-S	IgG_1_		U	
XBP1s	CHO-K1	IgG_1_	Prolonged viability, increased titer	U	
		EPO-Fc		U	
BLIMP1/XBP1s		IgG_1_	Decreased VCD, prolonged viability, increased titer, q_p_	U	
		EPO-Fc		U	
XBP1s	CHO-S	IgG_1_	Prolonged viability, increased titer	U	
		EPO-Fc		U	
BLIMP1/XBP1s		IgG_1_	Decreased VCD, prolonged viability, increased titer, q_p_	U	
		EPO-Fc		U	
BLIMP1α	CHO DG44	DTE Human Bone Morphogenetic Protein-4 (rhBMP-4)	Increased q_p_	U	[119]
BLIMP1β			Increased q_p_, yields	U	
	CHO-K1	ETE Rituximab	Decreased specific growth rate, increased titer, q_p_	U	
SCD1	CHO-K1d	cB72.3, FcFP, DTE IgG_1_	Increased titers	U	[42,141]
SREBF1				U	
PERK	CHO DG44	TNFR-Fc	Decreased aggregates	N	[94]
CERT	CHO DG44	Human Serum Albumin (HSA)	Increased titers, q_p_	U	[142]
		IgGs	Increased secretion	U	
XBP1s	CHO-K1/CHO-K1d	Secreted Alkaline Phosphatase (SEAP)	Increased production	U	[143]
		*Bacillus stearothermophilus*-derived a-amylase (SAMY)		U	
		Vascular Endothelial Growth Factor 121 (VEGF121)		U	
SRP14	CHO-K1	ETE Trastuzumab (Tras)	Prolonged viability, increased q_p_	U	[20]
		DTE Infliximab (Infli)	Increased q_p_	U	
SRP14/SRP9/SRP54/SR				U	
SRP14/SR/Translocon				U	
BIP	CHO-S	DTE Sp35Fc	Dose-dependent; Decreased IVCD, increased titer, q_p_	N	[19]
PDI			Increased titer, q_p_, product aggregation	Y	
CypB			Increased IVCD, titer, decreased product aggregation	N	
ATF6α			Dose-dependent; decreased IVCD, increased titer, q_p_	N	
XBP1s			Dose-dependent; decreased IVCD, increased titer, q_p_	N	
PDIA4	CAT-S/CHO-K1d	BsAb1	None	U	[144]
UBXN8			Decreased titer	U	
DNAJB9			None	U	
BIP			Decreased titer	U	
GRP94			Decreased product aggregation	N	
DNAJC3			None	U	
CHOP			Decreased product aggregation, titer	N	
HERPUD1			Decreased titer	U	
PDIA4	CHO-Sd	ETE Trastuzumab (Tras)	None	U	
UBXN8			None	U	
DNAJB9			None	U	
BIP			None	U	
GRP94			Increased titer	U	
DNAJC3			Increased titer	U	
CHOP			None	U	
HERPUD1			None	U	
PDIA3	CHO-DUKX B-11	Thrombopoietin (TPO)	Increased titer, q_p_	U	[145]
ERGIC-53	CHO-HcD6 (CHO-K1d)	IgG_1_	Increased VCD, titer, q_p_	N	[127]
ERGIC-53/MCFD2			Decreased VCD, increased titer, q_p_	N	
(CerS2/Tbc1D20)	CHO DG44	Human Serum Albumin (HSA) and IgG	Increased titer, q_p_	N	[146]
CHOP	CHO-S	hTRA-8	Increased titer	N	[128] ^#^
BIP	CHO-K1d	Multiple IgG_1_-type mAbs	Increased titer, q_p_ for one mAb	U	[147]
CypB			Increased cell growth, titer, decreased q_p_	U	
PDI			Increased titer, q_p_ for one mAb	U	
ATF6α			Increased titer, q_p_ dependent on expression level	U	
XBP1s				U	
(UBR4/UBR5)	-	IgG	Increased titer	U	[55]
EIF2AK2	CHO-S	DTE Thrombospondin 4 (THBS4)	Decreased titer	U	[148]
HSPA1B			None	U	
TBC1D9			None	U	
HSPA4L			None	U	
RAB11FIP1			Decreased titer	U	
MYO5B			None	U	
MGAT3			Decreased titer	U	
SNAP25			Decreased titer	U	
AGAP2			None	U	
RAB6B			None	U	
DERL3			Decreased titer	U	
SVIP1			Decreased titer	U	
GALNT18			Decreased titer	U	
JUN			Increased titer	U	
PDIA4			None	U	
ATF4			Increased titer	U	
SRP9			Increased titer	U	
HSPA8			None	U	
PDIA3			None	U	
RAB31			None	U	
RAB43			None	U	
HSPA1B		DTE Artemin (ARTN)	None	U	
ATF4			Increased titer	U	
SRP9			None	U	
PDIA3			Increased titer	U	
RAB43			Decreased titer	U	
HSPA8			Increased titer	U	
HsQSOX1b/Survivin	CHO-K1	Pembrolizumab (PAb)	Increased titer, q_p_	N	[129]
(CHOP)	-	TNFR-Fc	Decreased percentage of non-viable/apoptotic cells under ER stress conditions	U	[149]
CHOP			Increased percentage of non-viable/apoptotic cells under ER stress conditions	U	
Onco-tyrosine kinase receptor (KIT)	CHO-K1	Green Fluorescent Protein (GFP)-Fc	Increased titer	U	[107]
XBP1s	CHO-K1d	mAb-transient	Increased titer, q_p_	U	[130] ^##^
Light Chain/XBP1s			Increased titer, q_p_	U	
CRELD2			Increased titer, q_p_	U	
Light Chain/CRELD2			Increased titer, q_p_	U	
XBP1s/CRELD2			Increased titer, q_p_	U	
Light Chain/XBP1s/CRELD2			Increased titer, q_p_	U	
PDI		mAb-stable	Increased titer, q_p_	U	
ERO1α			Increased titer, q_p_	U	
PDI/ERO1a			Increased titer, q_p_	U	
SRP14			Increased titer, q_p_	U	
PDI/SRP14			Increased titer, q_p_	U	
ERO1α/SRP14			Increased titer, q_p_	U	
PDI/ERO1α/SRP14			Increased titer, q_p_	U	
CNX	-	TNFR-Fc	Increased q_p_	U	[108]
ATF6α	CHO-S	SEAP	Increased yield, q_p_	U	[150]
XBP1s			No increase in yield	U	
CypB			No increase in yield	U	
ERO1α			No increase in yield	U	
PDI			No increase in yield	U	
PDIA4			No increase in yield	U	
BIP			No increase in yield	U	
CRT			No increase in yield	U	
CNX			No increase in yield	U	
HSPA1A			No increase in yield	U	
TOR1A			No increase in yield	U	
CERT			No increase in yield	U	

* Targets in parentheses are downregulated or knockdown; XBP1 is induced by BLIMP1 in plasma cells, and BLIMP1 is therefore included in Table 3 [120]; other targets involved in the secretory pathway are also included; ** CHO-K1d refers to CHO-K1-derived host cell line; CHO-Sd refers to CHO-S-derived; CHO DHFR refers to dihydrofolate reductase deficient; DG44 is CHO DHFR-derived; *** Integral of viable cell density (IVCD); viable cell density (VCD); productivity (q_p_); **** (Y/N/U) for quality refers to (yes/no/unknown); “No” represents unaffected or improved; “Yes” represents negative impact; ^#^ Nishimiya et al. also perform additional co-expression studies with CHOP in COS-1 cells [128]; ^##^ Cartwright et al. also perform overexpression of multiple UPR biomarkers in the cell lines shown [130].
